# Near-Infrared Light and Solar Light Activated Self-Healing Epoxy Coating having Enhanced Properties Using MXene Flakes as Multifunctional Fillers

**DOI:** 10.3390/polym10050474

**Published:** 2018-04-26

**Authors:** Yuting Zou, Liang Fang, Tianqi Chen, Menglong Sun, Chunhua Lu, Zhongzi Xu

**Affiliations:** 1 State Key Laboratory of Materials-Oriented Chemical Engineering, College of Materials Science and Engineering, Nanjing Tech University, Nanjing 210009, China; zouyuting@njtech.edu.cn (Y.Z.); chentianqi@njtech.edu.cn (T.C.); sunmenglong1601@njtech.edu.cn (M.S.); zzxu@njtech.edu.cn (Z.X.); 2 Jiangsu Collaborative Innovation Center for Advanced Inorganic Function Composites, Nanjing Tech University, Nanjing 210009, China; 3 Jiangsu National Synergetic Innovation Center for Advanced Materials (SICAM), Nanjing Tech University, Nanjing 210009, China

**Keywords:** self-healing, epoxy coating, MXene, Diels-Alder reaction, photothermal effect

## Abstract

Two issues are required to be solved to bring intrinsically self-healing polymer coatings into real applications: remote activation and satisfied practical properties. Here, we used MXene, a newly reported two-dimensional material, to provide an epoxy coating with light-induced self-healing capabilities and we worked to enhance the properties of that coating. The self-healing coatings had a reversible crosslinking network based on the Diels-Alder reaction among maleimide groups from bis(4-maleimidopheny)methane and dangling furan groups in oligomers that were prepared through the condensation polymerization of diglycidylether of bisphenol A and furfurylamine. The results showed that the delaminated MXene flakes were small in size, around 900 nm, and dispersed well in self-healing coatings. The MXene flakes of only 2.80 wt % improved greatly the pencil hardness of the coating hardness from HB to 5H and the polarization resistance from 4.3 to 428.3 MΩ cm^−2^. The self-healing behavior, however, was retarded by MXene flakes. Leveling agent acted a key part here to facilitate the gap closure driven by reverse plasticity to compensate for the limitation of macromolecular mobility resulting from the MXene flakes. The self-healing of coatings was achieved in 30 s by thermal treatment at 150 °C. The efficient self-healing was also demonstrated based on the recovery of the anti-corrosion capability. MXene flakes also played an evident photothermal role in generating heat via irradiation of near-infrared light at 808 nm and focused sunlight. The healing can be quickly obtained in 10 s under irradiation of near-infrared light at 808 nm having a power density of 6.28 W cm^−2^ or in 10 min under irradiation of focused sunlight having a power density of 4.0 W cm^−2^.

## 1. Introduction

Self-healing polymer coatings have attracted academic and industrial interest in last several decades because their ability to recover aesthetic appearance and performance from a damaged status can greatly prolong service life and improve cost efficiency [1,2,3,4]. The widely investigated capsular and vascular healing mechanisms usually have the issues of limited healing times and expensive catalysts [5]. Therefore, the intrinsic self-healing mechanism of polymer coatings has also been paid great attention [6]. Upon stimulus, the reversible physical or chemical connections introduced in the polymer main structures dissociate to enable polymer chains to regain mobility. After gap closure and re-association of inherent reversible connections, damaged structures and properties around coating cracks are restored, leading to an efficient self-healing. To date, Diels-Alder (DA) reactions are widely reported to create versatile reversible crosslinking bonds in self-healing polymers [7,8,9,10]. At high temperature, the bonding between multi-furan groups and multi-maleimide groups dissociates, generating a mobile phase which flows into and fills the crack. Healing is completed after the reconnection of the fractured polymer parts at low temperature.

Two issues are required to be solved to bring intrinsically self-healing polymer coatings into real applications. Because direct heating is not a convenient approach for complex substrate geometry and outdoor usage, remote activation of intrinsic self-healing materials is of great importance for practical applications to achieve necessary and periodic maintenance [2,11]. Functional fillers that convert electrical field, magnetic field, and electromagnetic waves into heat, including metal-based [12] and carbon-based [6,12] filler systems, are incorporated into self-healing materials. The self-healing, thus, can be realized in a remote and precise manner. Moreover, there is a need to make a compromise between practical properties and intrinsically self-healing capabilities that usually require a mobile phase. The maintenance of coating usability is the bottom line to design new coating structures having inherent reversible connections. It has been a routine approach to improve fundamental properties of polymer coatings by various fillers [13,14,15,16,17], especially two-dimensional (2D) materials, including graphene [13], glass flake [14], and clay platelet [15]. Therefore, one hypothesis arises that the introduction of multifunctional fillers in intrinsic self-healing polymer coatings could not merely provide photothermal effects for remotely triggered self-healing, but enhance properties of polymer coatings as well.

A new 2D material called MXene has been a hot topic recently; it consists of transition metal carbides, nitrides, and carbonitrides [18,19,20,21]. MXene is usually produced via etching A layers of MAX phases which are layered ternary compound having a formula of M_n+1_AX_n_ (*n* = 1, 2, 3) [21]. A is predominantly an IIIA or IVA group element, M is the early d-block transition metal, and X is either C and/or N. After etching, MXene can be simply referred to as M_n+1_X_n_, with examples such as Ti_3_C_2_ and Ti_2_C, having surface groups of –F and/or –OH, etc. [22]. To date, a great deal of effort has been made to create delaminating multilayered MXene that presents excellent mechanical properties, thermal conductivity, and electrical conductivity [23,24]. In addition, MXene has been introduced to create novel polymer composites having enhanced properties [24,25]. For example, MXene was reported to act as the nucleation center for ultrahigh molecular weight polyethylene (UHMWPE) and increase the mechanical and creeping performance of the composites [26]. MXene-polyacrylamide nanocomposite films with great conductivity were also prepared [27]. Moreover, the capability of MXene to absorb light and microwaves has been reported as well, exploring its applications in tumor therapy as well as water evaporation and desalination [28,29]. Therefore, MXene is anticipated to be a novel candidate to play a dual role for remotely controlled self-healing polymeric coatings.

In our previous report, a thermally induced self-healing epoxy coating with fully reversible crosslinking networks was created based on DA reactions [30]. The results suggested that mechanical and electrochemical corrosion properties of such self-healing epoxy coating are comparable with conventional epoxy coatings with irreversible networks. Therefore, in the present work, MXene was used as the multifunctional filler to further improve the practical properties of the reported polymer coatings and to realize the remotely stimulation of the intrinsic self-healing. More specifically, MXene was delaminated and characterized before its effects on the mechanical and anti-corrosion properties of polymer coatings were investigated. Subsequently, the thermally-induced self-healing behaviors of the coatings containing MXene were examined. Finally, near-infrared light and sunlight were used to demonstrate the feasibility of self-healing in a remote manner.

## 2. Experimental

### 2.1. Materials

Diglycidylether of bisphenol A (DGEBA) and furfurylamine (FA) were obtained from Sigma Aldrich (St. Louis, MO, USA). Ti_3_AlC_2_ powders were purchased from Lianli New Technology Co., Ltd. (Beijing, China). Bis(4-maleimidopheny)methane (BMI) and tetrapropylammonium hydroxide (TPAOH, 25 wt % aqueous solution) were achieved from J&K Chemical (Beijing, China). *N*,*N*-dimethylformamide (DMF) was purchased from Aladdin (Shanghai, China). Leveling agent (XYS-5303) was purchased from New Element Chemical Co., Ltd. (Changzhou, China). All chemicals were used without further purification.

### 2.2. MXene Preparation and Delamination

Ten gram Ti_3_AlC_2_ powders were immersed in 100 mL hydrofluoric acid (HF) aqueous solution (49%) at 30 °C for 7 d. After centrifugation and washing by water and ethanol, the precipitates (named as un-delaminated MXene) were dispersed in 50 mL TPAOH under stirring for 3 d at room temperature, according to a previous report [29]. Then, the powders (named as delaminated MXene) were collected by centrifugation and rinsed five times using water and ethanol to remove the residual TPAOH.

### 2.3. Coating Preparation

An oligomer was required to be synthesized first via the reaction between DGEBA and FA, as reported previously [30]. In brief, 24 g DGEBA and 6.848 g FA were dissolved in DMF (40 g) in a sealed conical flask. The solution was heated at 100 °C using an oil bath. The reaction was finished in 6 h of constant stirring. The oligomer remained in DMF without precipitation, and the weight ratio was 43.5 wt %.

The oligomer/DMF solution (6.78 g, containing 2.95 g oligomer) and BMI (0.526 g) were mixed in a glass vial. A certain amount of delaminated MXene was dispersed by ultrasound in 1 mL DMF for 1 h, before being added into the above mixture and stirred vigorously. Leveling agent was also added as necessary. The mixture was carefully poured onto glass or steel plates located on the sample holder of a spin coater (KW-4A, Institute of Microelectronics of Chinese Academy of Science, Beijing, China), while the spin coating was performed at 400 rpm for 1 min. The curing was performed at 60 °C for 12 h in an oven. The bulk samples were prepared via pouring the mixture into a Teflon mold, and the same curing procedure was performed. The examined coating recipes and names are listed in Table 1.

### 2.4. Crack Formation

A Pencil Hardness Tester (H501-1, Elcometer, Manchester, UK) attached with a steel scalpel was used to create fresh cracks on the coating surface. The crack formation was obtained by moving the head along the coating surface slowly.

### 2.5. Characterizations

The structures of the un-delaminated and delaminated MXene as well as the coatings were characterized by X-ray diffraction (XRD, SmartLab-9KW, Rigaku, Japan) at a scan rate of 10° min^−1^ and 2θ ranging from 5° to 70°. Scanning electron microscope (SEM, JSM-5900, Japan Electronics Co., Ltd., Tokyo, Japan), transmission electron microscope (TEM, JEM-200CX, Japan Electronics Co., Ltd., Tokyo, Japan), and Raman spectra (LabRAMHR, HORIBA Ltd., Kyoto, Japan) having a laser wavelength of 532 nm were used to characterize MXene structures and morphologies. Particle size of delaminated MXene was evaluated by dynamic light scattering using Nanoplus (Micromeritics, Norcross, GA, USA). The chemical structures of the prepared epoxy resins and the reversibility of DA and retro-DA reactions were determined using Fourier-transform infrared spectroscopy (FTIR, Vector 22, Bruker, Billerica, MA, USA). The crosslinking density of the epoxy networks was assessed by swelling experiment. One set of the samples was stored in DMF for 24 h. The original mass of the sample was recorded as *m*_1_. After removing the sample from DMF, the sample weight (*m*_2_) was immediately measured in the swollen state after cleaning off extra solvent using a tissue. The swollen sample was heated at 60 °C until there was negligent weight variation and then the final weight (*m*_3_) was measured. The gel content (*G*) was given by *m*_3_/*m*_1_ × 100%. The swelling degree (*Q*) was calculated using Equation (1). The *ρ*_1_ and *ρ*_2_ were the specific densities of DMF and DGEBA.

(1) $$Q=\left[1+\rho_{2}\left(\frac{m_{2}}{\rho_{1}m_{1}}-\frac{1}{\rho_{1}}\right)\right]\times 100\%$$

Differential scanning calorimetry (DSC, STARe system, Mettler-Toledo, Zurich, Switzerland) was used to characterize the thermal behaviors of the samples. The scanning temperature ranged from room temperature to 150 °C, before it was cooled to −75 °C and then increased to 150 °C again. The heating and cooling rates were 10 °C min^−1^. Thermogravimetric analysis (TGA, STARe system, Mettler-Toledo, Zurich, Switzerland) was used to characterize the degradation temperature of the samples by increasing the temperature from room temperature to 800 °C at a heating rate of 10 °C min^−1^ in an air flow. Electrochemical workstation (CHI660E, Chenhua Instruments Co., Ltd., Shanghai, China) was used to carry out the Tafel analysis in a 3.5% NaCl solution on coated tinplate surfaces. The working, counter, and reference electrodes were sample, Pt, and saturated calomel electrode (SCE), respectively. The testing area was 1 cm^2^. The scan range was between −0.7 and −0.2 V at a scan rate of 1 mV s^−1^. According to standard test methods: pencil hardness GB/T 6739-96, adhesion GB/T 1720-89, and flexibility GB/T 1731-93, the mechanical properties of epoxy coatings on tinplate were assessed. Optical microscopy (BA210, Motic China Group Co., Ltd., Xiamen, China) was used to evaluate the self-healing behaviors of the cracks in the coating surface upon thermal treatments or light irradiation. The coatings were irradiated by near-infrared (NIR) light at 808 nm (FC-808-10W, Xinchanye Corp., Shenzhen, China) or solar light (Model 94043A, Newport, RI, USA) for 3 min before the balanced temperatures were measured using a handheld thermal imaging camera (HT-02, XINTEST Technology Co., Ltd., Shenzhen, China).

## 3. Results and Discussion

### 3.1. Structures and Morphologies of Delaminated MXene

The raw material used in the present work was a commercially available Ti_3_AlC_2_ layered MAX phase. Hydrofluoric acid (HF) aqueous solution was used to etch the Al layers. Such HF-treated MAX-phase powders are named as the un-delaminated MXene. Subsequently, the powders were treated by TPAOH for 72 h according to a previous report [29] and sonicated in the presence of DMF for 1 h. The as-treated powders are named as the delaminated MXene.

**Figure 1:**
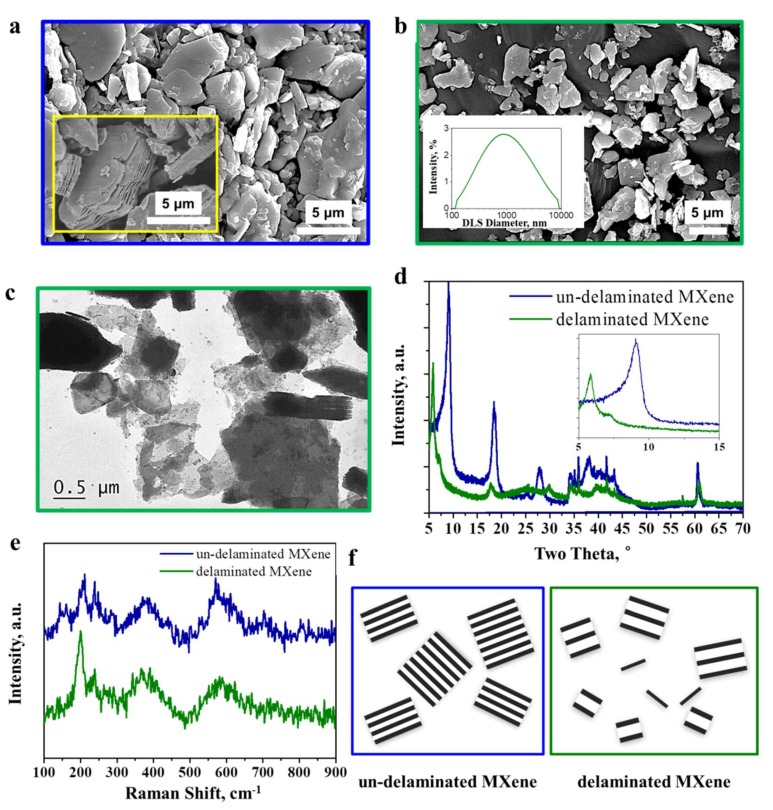
(**a**) SEM images of un-delaminated MXene; (**b**) SEM image of delaminated MXene and the particle size distribution (the inset); (**c**) TEM image of delaminated MXene; (**d**) XRD patterns of un-delaminated and delaminated MXene; (**e**) Raman spectra of un-delaminated and delaminated MXene; (**f**) Schematic diagram of the un-delaminated and delaminated MXene.

As shown in Figure 1a and the inset, the un-delaminated MXene of Ti_3_C_2_ exhibited multilayered structures having interlayer spaces, when Al layers in the MAX phase of Ti_3_AlC_2_ were removed via HF etching. The TPAOH intercalation and sonication evidently facilitated the transformation of the un-delaminated MXene sheet aggregates into thin and small flakes (Figure 1b). Such delaminated MXene presented sizes ranging from 115 nm–9 μm and a featured peak at 900 nm (the inset in Figure 1b). More importantly, the TEM image, as shown in Figure 1c, suggests that ultra-thin flakes existed in the delaminated MXene, possibly due to the exfoliation effect.

The XRD patterns of the un-delaminated and delaminated MXene are illustrated in Figure 1d. It is clear that the TPAOH and sonication treatment shifted the (002) peak from 2θ = 9.0° to 2θ = 5.8°. A sharp increase in lattice parameter *c* from 19.6 to 30.4 Å, thus, was achieved, suggesting the efficient intercalation. Besides, Figure 1e shows the Raman spectra of the un-delaminated and delaminated MXene, which agree very well with previous studies [31,32,33]. The Raman peaks located at 198 and 717 cm^−1^ are assigned to the A_1g_ symmetry out-of-plane vibrations of Ti and C atoms, while the peaks at 284, 366, and 624 cm^−1^ are the E_g_ group vibrations, including in-plane modes of Ti, C, and surface functional group atoms, respectively [31]. The combination of the results from SEM, TEM, DLS techniques, and XRD demonstrates that the size of the delaminated MXene flakes was reduced by the TPAOH treatment and sonication, while the interlayer spacing was increased (Figure 1f).

### 3.2. Structures and Properties of Epoxy Coatings Containing MXene

**Scheme 1:**
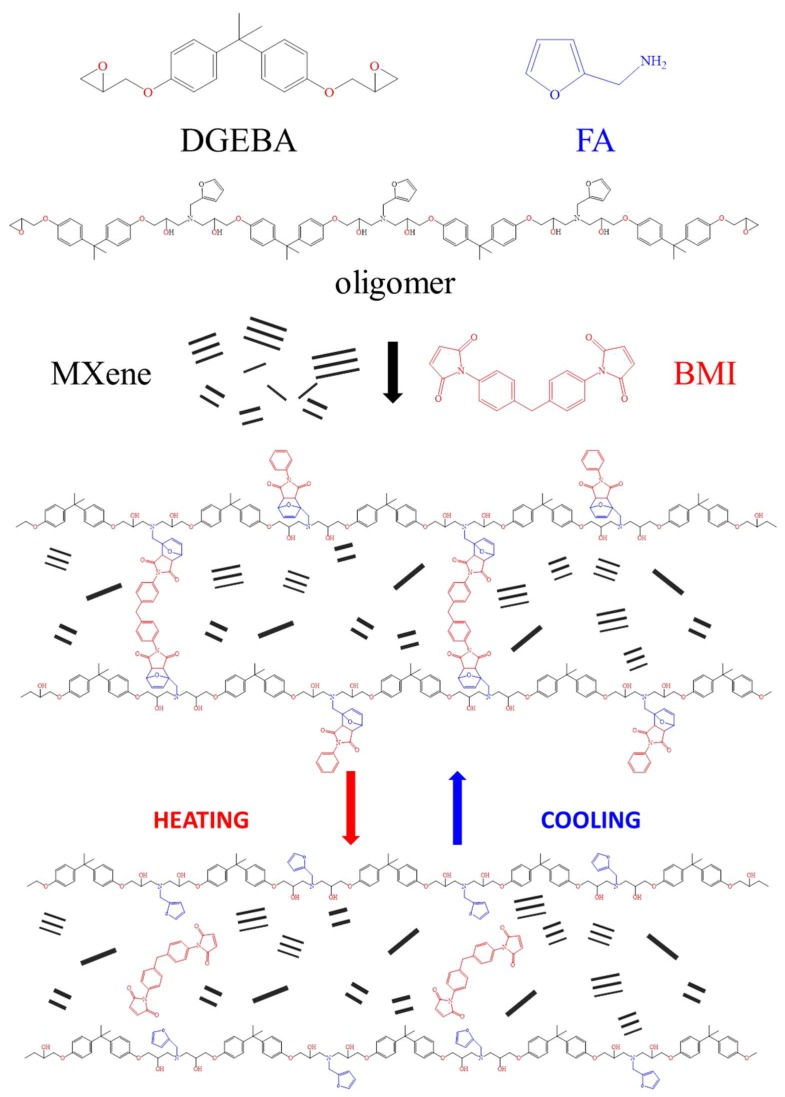
Chemical structures of DGEBA, FA, and synthesized oligomer as well as the variations of reversible networks upon heating and cooling due to retro-DA and DA reactions. DA = Diels-Alder.

To create the self-healing epoxy coatings containing MXene, an oligomer (*M*n = 5.9 kDa, GPC) that contained furan rings as dangling groups was prepared via the reaction between DGEBA and FA, according to our previous report [30]. As shown in Scheme 1, the oligomer solution was then mixed with BMI and delaminated MXene before the mixture was coated onto substrates and cured at 60 °C for 12 h. The DA reactions among furan rings and maleimide groups in the BMI created reversible networks which can dissociate at 150 °C (Scheme 1).

**Figure 2:**
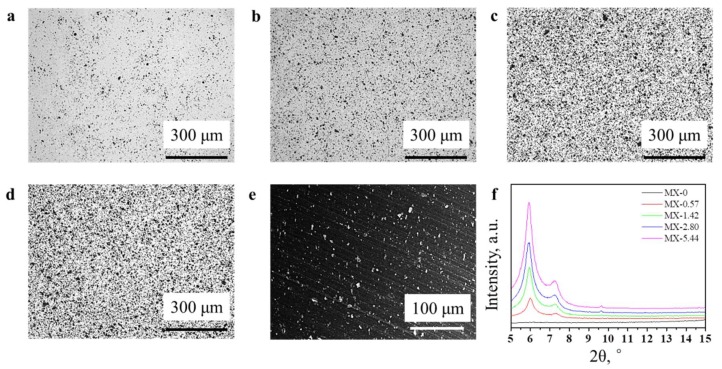
(**a**–**d**) Optical microscopy images of self-healing coatings containing MXene: (**a**) MX-0.57; (**b**) MX-1.42; (**c**) MX-2.80; and (**d**) MX-5.44; (**e**) SEM image of the fractured surface of MX-2.80; (**f**) XRD patterns of MX-0–MX-5.44.

The dispersion of the delaminated MXene in epoxy coatings was examined using an optical microscope. Because the light was irradiated upward from the bottom of the coated glass substrate, the black areas were assigned to MXene flakes dispersing in the coatings. As shown in Figure 2a, the MXene flakes dispersed very well in epoxy coatings at the loading of 0.57 wt %, and no evident agglomeration was observed. By increasing the MXene content to 1.42, 2.80, and 5.44 wt % (Figure 2b–d), good dispersion was still achieved. SEM characterization was used to further confirm the uniform dispersion of MXene flakes (Figure 2e) in MX-2.80. Therefore, without the need of any specific surface modification, MXene flakes are still compatible with the polymer matrix due to the existence of abundant polar groups. This is an advantage over other 2D materials. Subsequently, we used XRD to examine the variations in the interlayer spacing. As shown in Figure 2f, negligible change in the (002) peak location was observed, indicating that the solution mixing method may not result in further intercalation of MXene by macromolecular chains. Similar results were also demonstrated in a previous report where, via melt mixing, the polymer chains of UHMWPE did not intercalate into the stacks of MXene either [27]. This phenomenon is different from the widely reported polymer/clay nanocomposites [34,35].

**Figure 3:**
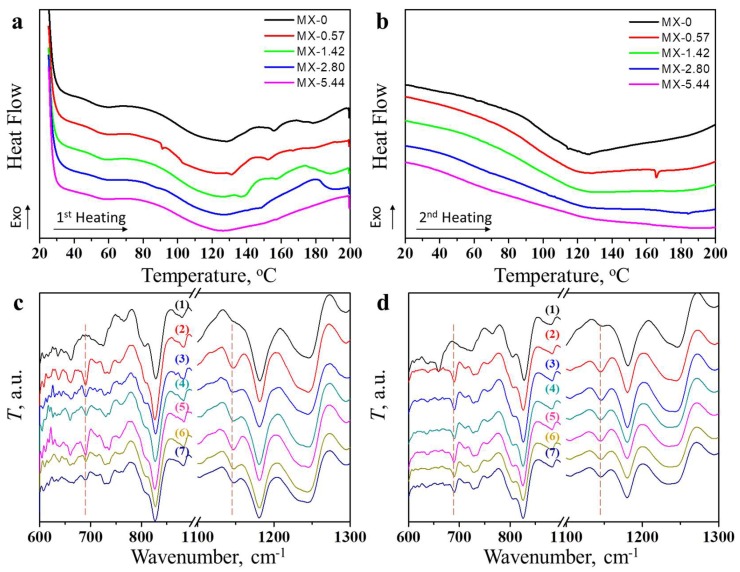
DSC heating curves of MX-0–MX-5.44: (**a**) first heating; (**b**) second heating; FTIR curves of (**c**) MX-2.80 and (**d**) MX-5.44 after different treatment: (1) after preparation; (2) heating at 150 °C for 10 min; (3) annealing at 60 °C for 1 h; (4) annealing at 60 °C for 2 h; (5) heating at 150 °C for 10 min; (6) annealing at 60 °C for 1 h; (7) annealing at 60 °C for 2 h. The vertical dashed lines indicate the locations of peaks at 696 and 1146 cm^−1^.

The effect of MXene content on the thermal properties of self-healing epoxy coatings was investigated next. As shown in Figure 3a, in the first DSC heating curves of MX-0–MX-5.44, evident endothermic peaks from 80 to 180 °C were all observed, which can be attributed to the retro-DA reaction. In other word, by increasing the temperature, the bonding between furan groups in epoxy oligomers and maleimide groups in BMI decoupled (Scheme 1). During the subsequent DSC cooling step, the DA reaction and the regeneration of reversible crosslinking points were expected. However, as shown in the second heating DSC curves (Figure 3b), the endothermic peak intensity gradually decreased as the MXene content was increased from 0 to 5.44 wt %. An obvious endothermic peak was still noticeable in MX-0, while the peak was negligible in MX-5.44. Although the cooling rate was as fast as 10 °C min^−1^, considerable DA reactions still occurred in MX-0. However, the regeneration of DA adducts was limited in MX-5.44, possibly due to the addition of delaminated MXene.

FTIR characterization is a good approach to evaluate the reversibility of DA reactions. As shown in Figure 3c, via heating MX-2.80 at 150 °C for 10 min, two evident peaks appeared at 696 and 1146 cm^−1^, which are assigned to the characteristic bands of maleimide and furan rings, respectively. This result indicates that the retro-DA reaction at 150 °C generated oligomers containing furan groups and released BMI (Scheme 1). The regeneration of DA adducts at a relatively low temperature was confirmed by the considerable reduction of peak intensities after the specimen was annealed at 60 °C for 1 and 2 h. The second heating and cooling treatment cycle resulted in the repeated increase and decrease in peak intensity, suggesting the reversibility of the crosslinking points. The similar variation trend of featured peaks was also observed in the MX-0 sample, as reported previously [30]. Therefore, it can be demonstrated that the small amount of MXene loading did not vary the reversibility. However, in MX-5.44, the intensities of the peaks at 696 and 1146 cm^−1^ did not vary during the repeated heating and cooling treatment. The increase in the content of delaminated MXene retarded the generation of DA-adducts, which agreed well with the DSC results.

**Figure 4:**
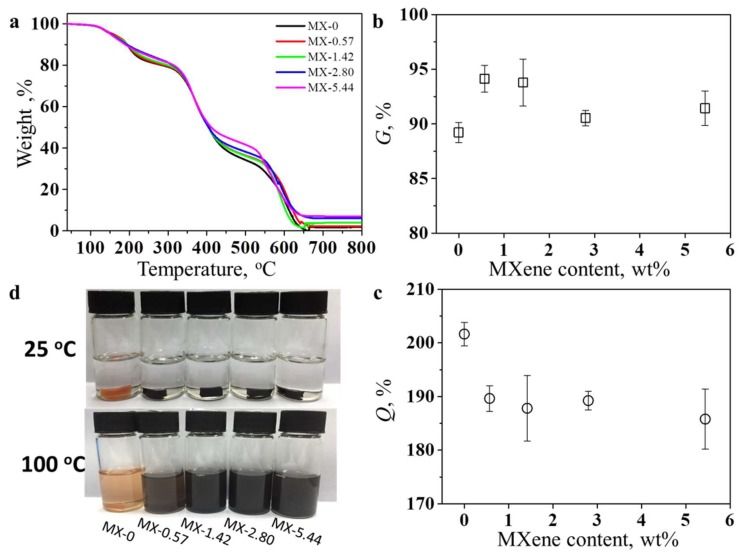
(**a**) TGA curves of MX-0–MX-5.44; (**b**) gel content and (**c**) swelling ratio of MX-0–MX-5.44; (**d**) Photographs of MX-0–MX-5.44 in DMF at 25 and 100 °C.

The influence of MXene on thermal degradation behaviors of the self-healing epoxy matrix was examined. As shown in Figure 4a, the addition of MXene flakes did not vary the evident three degradation steps, but retarded their initiation, i.e., the degradation temperature was increased via the addition of delaminated MXene. As listed in Table 2, *T*_d,15%_, indicating the temperature at 15% weight loss, increased greatly from 216 °C for MX-0 to 244 °C for MX-5.44. The temperature at 60% weight loss, *T*_d,60%_, also evidently shifted from 443 °C for MX-0 to 519 °C for MX-5.44. It is worth noting that the mass of the residuals after 650 °C were smaller than the theoretical values due to the mass loss of pristine MXene [27]. Figure 4b, c illustrate the variations of gel content (*G*) and swelling ratio (*Q*) with MXene content. The addition of MXene slightly increased *G* and reduced *Q* (Table 2). It is widely accepted that *G* and *Q* are related to crosslinking level of the polymer network, i.e., large *G* and small *Q* suggest a high crosslinking density. Therefore, it can be demonstrated that MXene flakes acted as physical crosslinking points to contribute to tense reversible networks. Moreover, the heating at 100 °C dissociated the reversible network and the oligomer dissolved in the solvent (Figure 4d).

**Figure 5:**
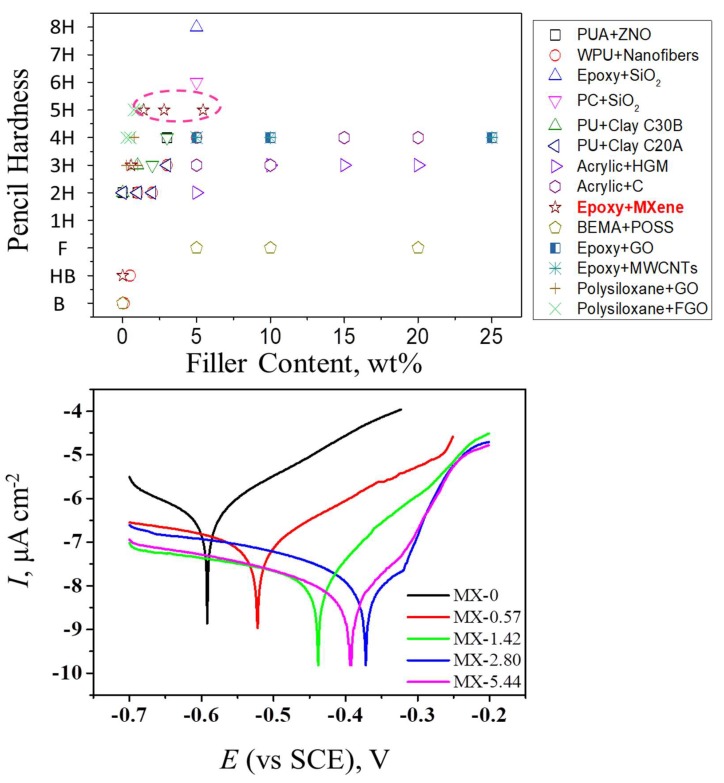
(**a**) Comparison of pencil hardness of different polymer coatings containing varied fillers; (**b**) Tafel plots of MX-0–MX-5.44.

In addition to the enhancement of thermal stability, the mechanical and anti-corrosion properties of the self-healing coatings were also improved via the addition of MXene. As shown in Table 2, the pristine MX-0 coating without MXene presented a pencil hardness of level B-HB. Only 0.57 and 1.42 wt % MXene flakes increased greatly the hardness to 2H-3H and 4H-5H, respectively. By increasing the MXene loading to 2.80 and 5.44 wt %, the hardness was not further improved. In addition, the adhesion and flexibility were not affected by MXene flakes, suggesting that the coatings maintained good bonding to the substrate and good toughness. Here, we roughly compared the pencil hardness of self-healing coatings having MXene flakes with other reported polymer coating systems containing different fillers. As shown in Figure 5a, ZnO nanoparticles (5 wt %) improved the hardness of polyurethane acrylic (PUA) coatings from 2H to 4H [36]. Good hardness was achieved for epoxy or polycarbonate (PC) coatings containing 5 wt % SiO_2_ [37], while polyhedral oligomeric silsequioxanes (POSS) of up to 20 wt % did not improve the hardness of bisphenol A ethoxylate dimethacrylate (BEMA) resin greatly [38]. Hollow glass microspheres (HGM) and cenospheres (C) of 20 wt % also increased slightly the hardness of acrylic coatings [39]. Moreover, anisotropic nanofibers of 5 wt % enhanced the surface hardness of waterborne polyurethane (WPU) coatings greatly from B to 4H [40], while two-dimensional clay or graphene flakes played a slight enhancing role for polyurethane (PU) [41], epoxy [42], and polysiloxane [43]. Moreover, the literature data show that (nano)particles noticeably improve the hardness of relatively soft polymer coatings [17,40] and medium-hard matrices [36,39,41,42,43,44], whereas their impact on discussed feature of intrinsically hard polymer layers is negligible [45,46]. In the present work, 1.42 wt % MXene flakes can greatly increase the hardness of self-healing epoxy coatings from HB to 5H, suggesting their critical role for practical applications.

Figure 5b shows the Tafel plot of self-healing coatings having varied MXene content to evaluate the effect on electrochemical corrosion properties. Generally, a high corrosion potential (*E*_corr_) and a low corrosion current (*I*_corr_) suggest a good corrosion protection on tinplate. As shown in Figure 5b and Table 2, *E*_corr_ increased and *I*_corr_ decreased by increasing MXene content up to 2.80 wt %. Further increasing MXene content to 5.44 wt % deteriorated the anti-corrosion capability. To further quantify the electrochemical corrosion properties, polarization resistance (*R_p_*) was calculated using the Stern-Geary equation [30],

(2) $$R_{p}=\frac{\beta_{a}\beta_{c}}{2.303\left(\beta_{a}+\beta_{c}\right)I_{\mathrm{corr}}}$$

where, *β_a_* and *β_c_* are the anodic and cathodic Tafel constants. The calculated *R_p_* is listed in Table 2 as well. By increasing MXene content from 0 to 2.80 wt %, the *R_p_* almost increased in an exponential manner from 4.3 to 428.2 MΩ cm^−2^, while *R_p_* of MX-5.44 decreased to 62.3 MΩ cm^−2^. The variation of such a critical parameter also suggests the great improvement in anti-corrosion capability of self-healing coatings via MXene flakes up to 2.80 wt %. When the tinplate was immersed in a neutral chloride solution, anodic and cathodic processes occurred. Fe became Fe^2+^ and OH^−^ was generated by O_2_ and H_2_O. Via such chemical oxidation processes, Fe_2_O_3_ finally generated on the tinplate surface. The existence of MXene flakes highly extended the diffusion length of H_2_O and O_2_ onto the metal surface and, thus, inhibited metal oxidation. Further increasing MXene flakes, however, might deteriorate the compactness of the coatings, leading to a direct diffusion path. In addition, MXene flakes up to 5.44 wt % intensified the corrosion reaction through the formed conductive networks in the coating matrix. Therefore, the further increased MXene reduced the anti-corrosion capability.

### 3.3. Thermally-Induced Self-Healing Capability

**Figure 6:**
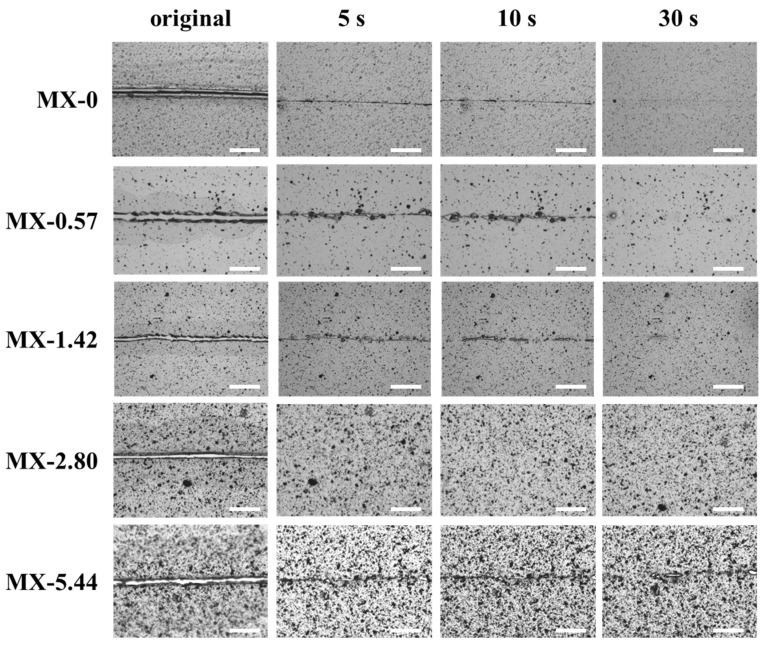
Optical images of MX-0–MX-5.44 coatings having cracks heating at 150 °C for different time periods. Scale bar = 100 μm.

Before light-triggered self-healing was investigated, we studied the thermally-induced self-healing behaviors of coatings containing MXene flakes. As shown in Figure 6, the gap having a width of 10 μm in MX-0 evidently closed after heating at 150 °C for 5 s, which then gradually disappeared in 30 s. The immediate gap closure in 5 s upon heating was possibly driven by the reverse plasticity. The reversible crosslinking points quickly dissociated upon further heating and “released” linear oligomers. The oligomers having high mobility diffused on the interface of the closed crack surface, healing the gap in 30 s. The DA reaction occurred at low temperature, gradually rebuilt the polymer network and promoted the recovery of coating properties. In the presence of a leveling agent, similar thermally-induced self-healing behaviors were observed for MX-0.57–MX-5.44. In these coatings containing MXene flakes, the evident gap closure occurred in 5 s as well before the cracks gradually disappeared in 30 s. The efficient thermally-induced self-healing, thus, can be achieved.

**Figure 7:**
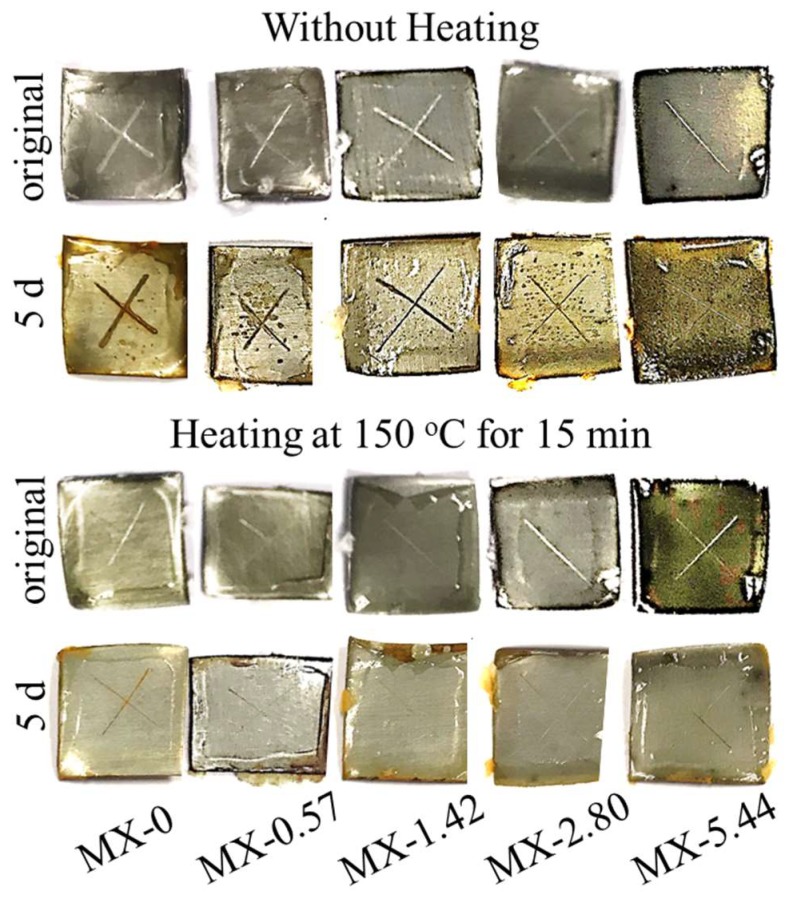
Photographs of MX-0–MX-5.44 coatings on tinplate before and after immersing in 3.5 wt % NaCl solution for 5 d.

In addition to the morphological observation, we evaluated the self-healing behaviors by assessing the variations in their corrosion protection performance. As shown in Figure 7, when MX-0–MX-5.44 coatings having evident cracks were directly immersed in 3.5 wt % NaCl solution for 5 d, severe corrosions were found on tinplate. Similar experiments were performed, while the coatings were heated at 150 °C for 15 min before immersing in NaCl solution. Due to the efficient crack closure and healing, minimal corrosions occurred on the surface of MX-0–MX-5.44 coatings.

**Figure 8:**
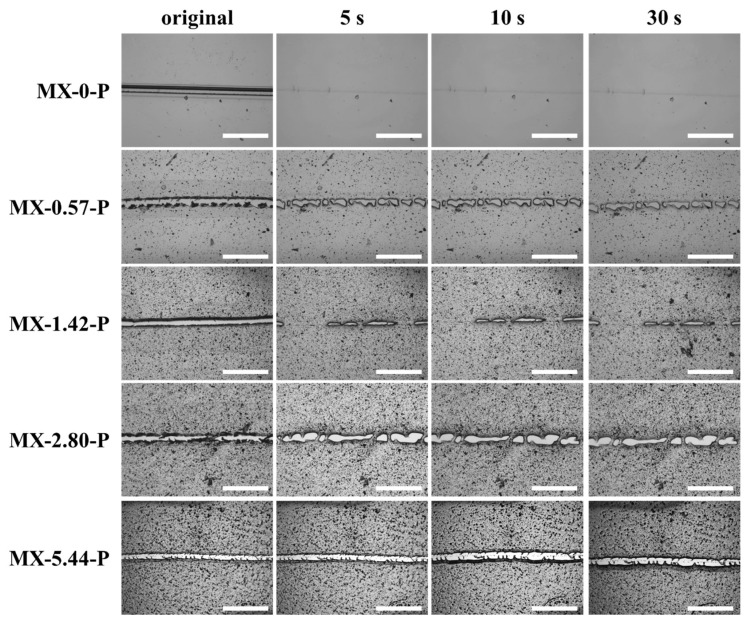
Optical images of MX-0-P–MX-5.44-P coatings having cracks heated at 150 °C for different time periods. No leveling agent was used. Scale bar = 300 μm.

There is a strong need to demonstrate the role of MXene flakes in self-healing without the aid of a leveling agent. We prepared such coatings, accordingly named MX-0-P–MX-5.44-P. As shown in Figure 8, without leveling agent and MXene flakes, MX-0-P still presented efficient self-healing behaviors in 30 s. The MXene flakes, however, greatly deteriorated the thermally-induced self-healing capability. The gaps having widths of 25–35 μm in MX-0.57-P–MX-5.44-P partially closed in 5 s, while the cracks were still visible upon further heating. It can be speculated that the existence of MXene flakes actually reduced the capability of gap closure driven by reverse plasticity due to their role in limiting the mobility of molecular chains. In comparison, the leveling agent to some extent counteracted this effect and promoted the gap closure (Figure 6).

In addition to reverse plasticity, surface tension driven viscoelastic flow, which is considered popularly as leveling, can drive the gap closure as well. However, according to our previous report [30], the dissociated oligomers did not wet the substrate well, and the gap closure of the prepared self-healing coating resulting from automatic leveling did not occur. Here, whether the leveling agent can promote the gap closure driven by surface tension was investigated as well. We created wide cracks of 250–400 μm on neat MX-0 and MX-0-P coatings as well as observed their gap closure behaviors upon heating, because the reserve plasticity can only work well for narrow cracks. The results are shown in Figure S1 (Supporting Information), and it is clear that the gap width did not reduce even after the addition of a leveling agent. This suggests that the role of the leveling agent in the self-healing behavior here was only to compensate for the role of MXene flakes and to promote the gap closure driven by reverse plasticity.

### 3.4. Light-Induced Self-Healing

**Figure 9:**
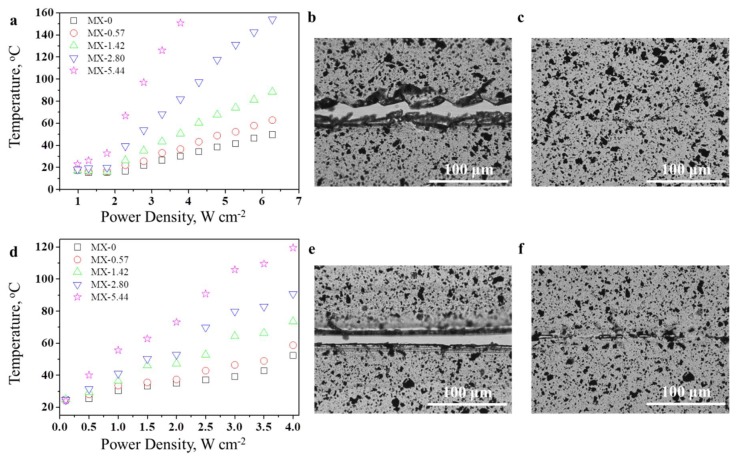
(**a**) Relation between temperature and 808 nm near-infrared (NIR) light power density; (**b**,**c**) Optical images of MX-2.80 having a crack (**b**) before and (**c**) after irradiation at 6.28 W cm^−2^ for 10 s; (**d**) Relation between temperature and focused sunlight power density; (**e**,**f**) Optical images of MX-2.80 having a crack (**e**) before and (**f**) after irradiation at 4 W cm^−2^ for 10 min.

It is important to achieve the remotely activated self-healing of polymer coatings because the direct heating is inconvenient in practical applications. MXene has been reported as a good photothermal filler for both near-infrared (NIR) light [29] and solar light [28]. Therefore, we investigated the photothermal effects of coatings containing MXene flakes and their light induced self-healing behaviors. As shown in Figure 9a, the temperature increased greatly with MXene content and power density of NIR light at 808 nm. For example, under irradiation at 3.28 W cm^−2^ for 3 min, the temperature of MX-0 was only 26.6 °C, which increased to 33.1, 43.4, 68.4, and 125.9 °C for MX-0.57, MX-1.42, MX-2.80, and MX-5.44, respectively. We investigated the light-induced self-healing behaviors of MX-10 for an example. As shown in Figure 9b,c, a crack having a width of ~22 μm on MX-2.80 quickly disappeared in 10 s after the irradiation with NIR light with a power density of 6.28 W cm^−2^. The quick crack closure and disappearance similar to a welding process upon irradiation is also shown in Supporting Information Video S1.

Sunlight provides a more convenient light source than NIR light for self-healing outdoors. As shown in Figure 9d, focused sunlight can also greatly increase the temperature of epoxy coatings having MXene flakes. For example, at 4 W cm^−2^, the temperatures of MX-0, MX-0.57, MX-1.42, MX-2.80, and MX-5.44 were 52.3, 58.7, 73.4, 90.7, and 119.4 °C, respectively. Similarly, under irradiation of focused sunlight at 4 W cm^−2^ for 15 min, self-healing was achieved for MX-2.80 (Figure 9e,f).

## 4. Conclusions

Two dimensional MXene flakes after delamination were used to enhance the properties of a synthesized coating with reversible networks based on Diels-Alder reactions and to achieve the light-induced self-healing. TEM, SEM, DLS, and XRD results suggested that the size of the delaminated MXene flakes was reduced to 900 nm by TPAOH treatment and sonication, while the interlayer spacing was increased to 30.4 Å. The delaminated MXene flakes dispersed well in the coatings, while the interlayer spacing was not further increased. The prepared MXene flakes of only 2.80 wt % improved greatly the coating hardness from HB to 5H and the polarization resistance from 4.3 to 428.3 MΩ cm^−2^. The self-healing behavior, however, was retarded by MXene flakes because of their limitation on macromolecular mobility during gap closure driven by reverse plasticity. Leveling agent was used to play the key role in facilitating the self-healing in 30 s by thermal treatment at 150 °C. The efficient self-healing was also proved by the recovery of anti-corrosion capability. Moreover, evident photothermal effects can be achieved by the MXene flakes via irradiation of near-infrared light at 808 nm and focused sunlight. The healing was quickly obtained in 10 s under irradiation of near-infrared light at 808 nm having a power density of 6.28 W cm^−2^ or in 10 min under irradiation of focused sunlight having a power density of 4.0 W cm^−2^. This work provides a feasible approach to provide satisfied properties together with remote-triggered self-healing capability for polymer coatings. Further work will include the design of new polymer coatings having good properties and the capacity to heal wide cracks in a remote manner.

## Figures and Tables

**Table 1:** Name and recipe of prepared coatings.

Name ^a^	Oligomer, g ^b^	BMI, g ^c^	MXene, g ^d^	Leveling Agent, g
MX-0	2.95	0.526	0	0.035
MX-0.57	2.95	0.526	0.02	0.035
MX-1.42	2.95	0.526	0.05	0.035
MX-2.80	2.95	0.526	0.10	0.035
MX-5.44	2.95	0.526	0.20	0.035

^a^: MX-0–MX-5.44 stand for coatings containing both MXene and leveling agent, while no leveling agent was included in MX-0-p–MX-5.44-p as shown later. ^b^: The oligomer/*N*,*N*-dimethylformamide (DMF) solution of 6.78 g, containing 2.95 g oligomer, was used to create self-healing coatings via spin coating: 2.95 g oligomer comprised of 1 g Diglycidylether of bisphenol A (DGEBA) and 1.95 g furfurylamine (FA) moieties in main chains in theory. ^c^: Bis(4-maleimidopheny)methane (BMI) was calculated to consume all furan groups in FA moieties in 2.95 g oligomer. ^d^: MXene was given as 2, 5, 10, and 20 phr of the 1 g DGEBA moieties in 2.95 g oligomer, contributing to 0.57, 1.42, 2.80, and 5.44 wt % of the whole coating matrix, respectively, without considering leveling agent.

**Table 2:** Thermal, mechanical, and electrochemical corrosion properties of MX-0–MX-5.44.

Properties	Thermo-Degradation Property		Mechanical Property			Electrochemical Corrosion		
Samples	*T*_d,15%_	*T*_d,60%_	Pencil Hardness	Flexibility	Adhesion	*E*_corr_	*I*_corr_	*R*_p_
	°C	°C	N/A	mm	N/A	mV	μA cm^−2^	MΩ cm^−2^
MX-0	216	443	B-HB	<2	1	−590	0.4514	4.3
MX-0.57	220	454	2H-3H	<2	1	−520	0.1030	10.4
MX-1.42	227	452	4H-5H	<2	1	−439	0.0151	76.5
MX-2.80	249	473	4H-5H	<2	1	−371	0.0047	428.2
MX-5.44	244	519	4H-5H	<2	1	−392	0.0382	62.3

## Supplementary Materials

The following are available online at http://www.mdpi.com/2073-4360/10/5/474/s1.
