# Supplementary material for: Near-Infrared Light and Solar Light Activated Self-Healing Epoxy Coating having Enhanced Properties Using MXene Flakes as Multifunctional Fillers
Source: Polymers (Basel). 2018 Apr 26;10(5):474. doi: 10.3390/polym10050474 (PMC6415427; doi:10.3390/polym10050474)
Supplement: Supplementary file 1 [file polymers-10-00474-s001.zip › supporting information/Supporting information.docx]

**Supporting Information**

Near-Infrared Light and Solar Light Activated Self-Healing Epoxy Coating having Enhanced Properties Using MXene Flakes as Multifunctional Fillers

**Yuting Zou ^1,2,3^, Liang Fang ^1,2,3,^*, Tianqi Chen ^1,2,3^, Menglong Sun ^1,2,3^, Chunhua Lu ^1,2,3,^* and Zhongzi Xu ^1,2,^****^3^**

^1^ State Key Laboratory of Materials-Oriented Chemical Engineering, College of Materials Science and Engineering, Nanjing Tech University, Nanjing 210009, China; zouyuting@njtech.edu.cn (Y.Z.); chentianqi@njtech.edu.cn (T.C.); sunmenglong1601@njtech.edu.cn (M.S.); zzxu@njtech.edu.cn (Z.X.)

^2^ Jiangsu Collaborative Innovation Center for Advanced Inorganic Function Composites, Nanjing Tech University, Nanjing 210009, China

^3^ Jiangsu National Synergetic Innovation Center for Advanced Materials (SICAM), Nanjing Tech University, Nanjing 210009, China

***** Correspondence: lfang@njtech.edu.cn (L.F.); Chhlu@njtech.edu.cn (C.L.); Tel.: +86-025-8358-7270 (L.F. & C.L.)

**Supporting information Figure S1:**


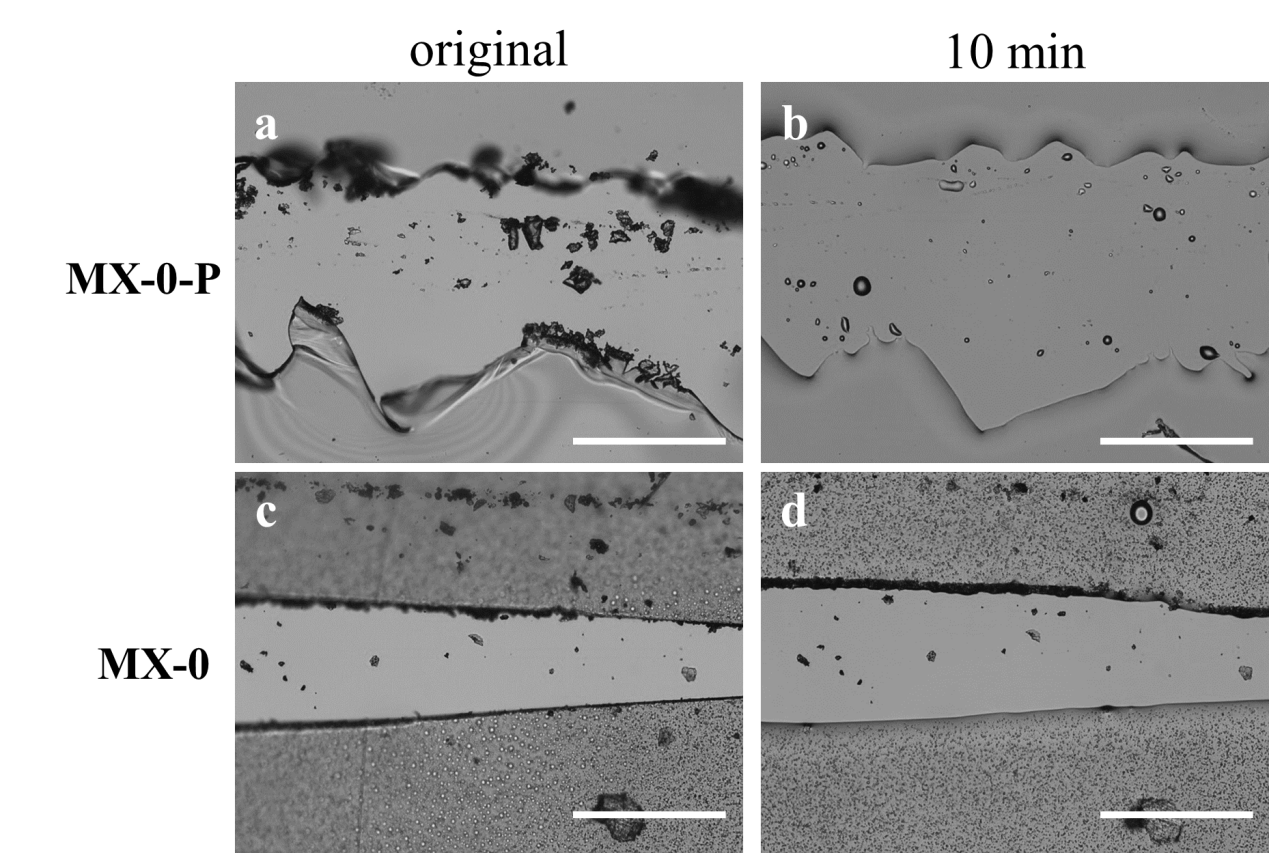


Figure S1. Optical image of MX-0-p and MX-0 coatings having wide cracks before and after heating.

**Supporting information Video:**

We used optical microscope (BA210, Motic China Group Co., Ltd., Xiamen, China) to observe and record the quick crack healing of MX-2.80 coating upon near-infrared (NIR) light irradiation having power density of 6.28 W cm^-2^. The coating having the original crack with width of ~20 μm was placed onto the sample stage of the microscope and moved back and forth to record its healing behavior before and after light irradiation.
